# Influence of illness perception on depression and anxiety in individuals with schizophrenia during the COVID-19 pandemic

**DOI:** 10.3389/fpubh.2025.1583630

**Published:** 2025-05-15

**Authors:** Meng Zhang, Yan-Feng Deng

**Affiliations:** Department of Psychiatry, Minhang District Mental Health Center, Shanghai, China

**Keywords:** illness perception, depression, anxiety, COVID-19, schizophrenia

## Abstract

**Objective:**

This study aimed to investigate the factors influencing depression and anxiety in individuals with schizophrenia during the COVID-19 pandemic, with the goal of providing evidence to support strategies for improving emotional disturbances during the pandemic.

**Methods:**

A cross-sectional study was conducted among 159 outpatients with schizophrenia between January and June 2023. Data were collected using a self-developed sociodemographic questionnaire, the Chinese version of the Illness Perception Questionnaire-Revised (IPQ-R), the Patient Health Questionnaire-9 (PHQ-9), and the 7-item Generalized Anxiety Disorder scale (GAD-7). Regression analysis was performed using SPSS version 27.0 to identify the factors associated with depression and anxiety.

**Results:**

Timeline cyclical and treatment control were significant predictors of depression among individuals with schizophrenia (R^2^ = 0.160, *p* < 0.01). Additionally, timeline cyclical and the presence of chronic comorbidities significantly predicted anxiety (R^2^ = 0.217, *p* < 0.01).

**Conclusion:**

Illness perception concerning COVID-19 significantly influenced the severity of depression and anxiety in individuals with schizophrenia. Addressing these perceptions serves as an essential component of interventions aimed at improving mental health outcomes during the pandemic.

## 1 Introduction

In January 2020, the outbreak of coronavirus disease 2019 (COVID-19) rapidly evolved into a global pandemic. During the COVID-19 pandemic, a significant increase on COVID-anxiety syndrome (1, 2), loneliness (3), distress (4), and a significant decrease on well-being (5) has been observed. This includes the issue of a significant increase in the prevalence of depression and anxiety among individuals with schizophrenia. A large-scale survey conducted in China reported that all community-dwelling individuals with schizophrenia experienced varying degrees of depression and anxiety during the pandemic (6). Among these individuals, 34.9% exhibited moderate to severe depression, while 16.9% experienced moderate to severe anxiety. Another study found that the prevalence of depression rose by 42.9%, and the prevalence of anxiety increased by 10.2% during the pandemic (7).

Illness perception refers to the process by which both patients and those without medical conditions, interpret and analyze symptoms or diseases based on their prior knowledge and experiences (8). This concept is rooted in the Self-Regulation Model (SRM), which posits that patients adopt coping strategies and assess their effectiveness based on their cognitive and emotional representations of the disease (9). Information derived from these assessments is subsequently used to modify future coping behaviors (10). In 1996, Weinman et al. developed the Illness Perception Questionnaire (IPQ) based on the foundational structure of the SRM (11). This questionnaire was later revised by Moss-Morris et al. to create the Illness Perception Questionnaire-Revised (IPQ-R), which demonstrated improved reliability and validity (12–15).

Additionally, Chinese researchers validated the reliability and validity of the Chinese version of the IPQ-R in studies involving patients with stroke, breast cancer, and obstructive sleep apnea syndrome (OSAS) (16–18).

Patients exhibiting depression-associated characteristics—such as reduced energy levels, impaired attention, and heightened feelings of despair—are less likely to adhere to recommended preventive measures during the COVID-19 pandemic (19). This nonadherence exacerbates their condition, potentially leading to a vicious cycle of worsening mental health. Several studies demonstrated a significant association between illness perception and severity of depression and anxiety (20–22). Based on the hypotheses derived from the Self-Regulation Model, a mediational model has been proposed in which coping mediates the effect of illness perception on health outcomes, including psychological distress (23). Moreover, many studies have verified this mediation process (20, 22, 24), among which a study even applied meta-analytic path analyses to corroborate this model (24), so it is concluded that illness perception and symptoms of depression and anxiety are associated through the mediation of coping. However, there is a lack of research examining the relationship between illness perception of COVID-19 and the presence of depressive and anxiety symptoms in individuals with schizophrenia.

The present study aims to examine this relationship, with the goal of identifying strategies to improve negative emotional responses and providing guidance on improving the psychological well-being of individuals with schizophrenia during the pandemic.

## 2 Participants and methods

### 2.1 Participants

Individuals with schizophrenia who sought care at the Minhang Mental Health Center in Shanghai between January and June 2023 were recruited using convenience sampling. The inclusion criteria were as follows: (1) a diagnosis of schizophrenia based on the criteria outlined in the tenth edition of the International Classification of Diseases (ICD-10), (2) an age range between 18 and 65 years, (3) completion of at least a primary level of education, and (4) a Positive and Negative Syndrome Scale (PANSS) score of ≤ 5 points for delusion, hallucinatory behaviors, exaggeration, suspiciousness/paranoia, and unusual thought content, with a PANSS score of ≤ 4 points for conceptual disorganization.

Exclusion criteria were: (1) a history of substance abuse or other mental disorders within the past 6 months, (2) a diagnosis of serious physical illnesses such as chronic obstructive pulmonary disease (COPD) or coronary heart disease (CHD), and (3) communication or reading disorders.

Informed consent was obtained from all participants. Based on these criteria, a total of 159 participants were enrolled in the study.

### 2.2 Evaluation tools

A cross-sectional survey method was used in this study, by having multiple questionnaires, including a self-developed sociodemographic questionnaire, the Chinese version of the IPQ-R, the Patient Health Questionnaire-9 (PHQ-9), and the 7-item Generalized Anxiety Disorder scale (GAD-7).

The Chinese version of the IPQ-R was used to assess the illness perception of COVID-19 by the participants. The IPQ-R consists of three sections. For this study, only the illness representation (IR) dimension, which includes 38 items from the second section, was selected to evaluate cognitive and emotional representations of COVID-19. This dimension is divided into seven subscales, and the definition of each is as follows. Timeline refers to the patient’s perceptions of the likely duration of illness, categorized as acute/chronic, and the predictability and variability of illness, categorized as cyclical; consequences are the patient’s beliefs regarding the illness severity and the likely influence on individual social and psychological functioning; personal control reflects the patient’s belief about the ability of taking measures to personally control the illness and self-efficacy; treatment control is the patient’s belief about the degree to which his or her condition is amenable to cure; illness coherence refers to the extent of patients’ understanding of their illness; and emotional representations are the patient’s general mood in response to the illness. Each item is rated on a five-point Likert-type scale (1 = strongly disagree, 5 = strongly agree). The item responses are summed to obtain the IPQ-R subscale scores.

The questionnaire used in this study was adapted to align culturally with the Chinese context and demonstrated higher reliability and validity than the original version when applied to outpatient populations in general hospitals (25). In this study, the Cronbach’s *α* coefficient for the IR dimension was 0.8, while the coefficients for the seven subscales ranged from 0.61 to 0.87.

In this study, the PHQ-9 and GAD-7 were used to assess depression and anxiety, respectively. The PHQ-9 is a self-assessment tool comprising of nine items designed to evaluate depressive symptoms over the preceding 2 weeks. Each item is scored on a scale from 0 to 3, with higher scores indicating greater severity of depression.

The GAD-7 is a self-administered scale consisting of seven items, intended to screen for and assess the severity of anxiety symptoms in the past 2 weeks. Similar to the PHQ-9, each item on the GAD-7 is scored from 0 to 3, with higher scores reflecting more severe anxiety symptoms.

In this study, the PHQ-9 demonstrated a Cronbach’s *α* coefficient of 0.880, while the GAD-7 exhibited 0.922.

### 2.3 Research process

The study was carried out in the Outpatient Department of Minhang Mental Health Center in Shanghai. One-on-one interviews were conducted with all participants. Initially, psychiatric symptoms were assessed using the PANSS. Participants who met the inclusion criteria were then instructed to independently complete the remaining questionnaires. During this process, explanations were provided by the research team only when participants sought clarification regarding specific items.

Informed consent was obtained from all participants prior to their inclusion in the study. The research protocol was reviewed and approved by the hospital’s Ethics Committee (Approval No.: LW202205).

### 2.4 Statistical analysis

Data analysis was conducted using SPSS for Windows, version 27.0 (SPSS 27.0). The internal consistency reliability of the Chinese versions of the IPQ-R, PHQ-9, and GAD-7 were assessed.

The normality of all data was evaluated using the Shapiro–Wilk test. Data following a normal distribution were expressed as mean ± standard deviation (^−^x ± s), analyzed using Pearson correlation, and compared between groups using the t-test or among groups using one-way analysis of variance (ANOVA). Data not following a normal distribution were expressed as the median (interquartile range), analyzed using Spearman correlation, and compared between groups using the Mann–Whitney U test or among groups using the Kruskal-Wallis H test.

Multiple linear regression analysis was used to examine predictive relationships between variables. A significance level of *p* < 0.05 was considered statistically significant.

## 3 Results

### 3.1 Description of the sample

Among the 159 participants included in the final sample, 58.5% were male, 67.3% were classified as young adults, 69.2% had an educational qualification of senior high school or higher, and 58.5% were unmarried. Only a small proportion of participants reported having chronic diseases. No significant differences were observed in demographic variables—such as age, education, occupation, and marital status—between those included in the sample and individuals who were excluded. Detailed demographic data are presented in Table 1.

**Table 1:** Sociodemographic data of participants.

Variables	N	%
Sex		
Male	93	58.5
Female	66	41.5
Age		
18–44 years	107	67.3
45–59 years	46	28.9
≥ 60 years	6	3.8
Education		
Junior high school or below	49	30.8
Senior high school or technical secondary school	61	38.4
Junior college or above	49	30.8
Marital status		
Unmarried	93	58.5
Married	34	21.4
Divorced or widowed	32	20.1
Combination of chronic diseases		
Yes	42	26.4
No	117	73.6

### 3.2 Illness perception of COVID-19

The quartile and Cronbach’s *α* coefficients for each subscale in the IR dimension are presented in Table 2. The results indicated that participants generally perceived the course of COVID-19 to be short and not likely to result in severe negative consequences. Most participants believed that personal efforts and medical treatment could improve their condition to some extent. Additionally, they reported a good level of understanding of the illness. Although many participants acknowledged the possibility of recurrence, they did not exhibit significant negative emotional responses when confronted with COVID-19. The Cronbach’s *α* coefficients for all subscales were within the ideal range.

**Table 2:** Quartile and Cronbach’s α coefficient of the illness representation (IR) subscale scores.

Subscale	Item number	*M* (*Q*1, *Q*3)	Cronbach’s α coefficient
Timeline (acute/chronic)	6	2.33 (2.00, 2.83)	0.70
Consequences	6	2.33 (2.00, 3.33)	0.85
Personal control	6	3.33 (3.00, 3.83)	0.61
Treatment control	5	4.00 (3.60, 4.20)	0.65
Illness coherence	5	2.40 (2.00, 3.20)	0.81
Timeline cyclical	4	2.50 (2.00, 3.00)	0.67
Emotional representations	6	2.33 (2.00, 3.33)	0.87

### 3.3 Correlation analysis between depression, anxiety and illness perception

The PHQ-9 score demonstrated a positive correlation with several domains of the IPQ-R, including consequences, illness coherence, timeline cyclical, and emotional representations (*p* < 0.05). Conversely, a negative correlation was observed between the PHQ-9 score and treatment control (*p* < 0.05). The GAD-7 score was positively correlated with the same IPQ-R domains—consequences, illness coherence, timeline cyclical, and emotional representations (*p* < 0.05).

Additionally, a significant correlation was found between the combination of chronic diseases and the GAD-7 score (*p* < 0.05), indicating that individuals with chronic diseases were more susceptible to anxiety. Further details are provided in Table 3.

**Table 3:** Variables correlated with PHQ-9 and GAD-7 scores.

Dependent variables	Correlated variables	r/U	*p*
PHQ-9 score	Consequences	0.218	0.015
	Illness coherence	0.295	0.001
	Timeline cyclical	0.294	0.001
	Emotional representations	0.330	<0.001
GAD-7 score	Treatment control	−0.202	0.024
	Consequences	0.286	0.001
	Illness coherence	0.259	0.004
	Timeline cyclical	0.390	<0.001
	Emotional representations	0.388	<0.001
	Combination of chronic diseases	1,049	0.049

### 3.4 Linear regression analysis of depression and anxiety

Linear regression analysis was conducted using the PHQ-9 and GAD-7 scores as dependent variables. Independent variables that revealed significant correlations with depression and anxiety in the univariate analysis were included in the model. The results indicated that the illness perception domains of timeline cyclical and treatment control were significant predictors of depression in individuals with schizophrenia during the COVID-19 pandemic (*R*^2^ = 0.160, *p* < 0.01). Specifically, timeline cyclical was identified as a risk factor for depression (*p* < 0.05), while treatment control served as a protective factor (*p* < 0.05).

For anxiety, timeline cyclical and the combination of chronic diseases were significant predictors (R^2^ = 0.217, *p* < 0.01). Both predictors were identified as risk factors for anxiety (*p* < 0.01). Further details are provided in Table 4.

**Table 4:** Multiple linear regression analysis of the PHQ-9 and GAD-7 scores.

Dependent variables	Predictive variables	*B*	*β*	*p*
PHQ-9 score	Treatment control	−1.448	−0.187	0.047
	Timeline cyclical	1.369	0.223	0.027
GAD-7 score	Combination of chronic diseases	2.644	0.242	0.006
	Timeline cyclical	1.713	0.288	0.003

## 4 Discussion

COVID-19 continues to circulate globally, exhibiting recurrent fluctuations approximately every 6 months. In China, it has transitioned into a periodically prevalent respiratory disease, with a proportion of cases developing severe symptoms. Individuals with mental health conditions, including those with schizophrenia, have been shown to have an elevated mortality risk from infectious diseases (26). Adherence to recommended preventive measures is often challenging for individuals with schizophrenia due to the influence of psychiatric symptoms.

Depression and anxiety are key factors influencing the likelihood of an individual adhering to COVID-19 preventive measures (19, 27). Therefore, understanding the factors contributing to depression and anxiety in individuals with schizophrenia during the pandemic is crucial for improving adherence to preventive measures during outbreaks of infectious diseases.

The study findings indicate that participants were less likely to develop depression if they believed that current treatments could effectively control COVID-19 infection. However, those who perceived COVID-19 as a recurring condition demonstrated a higher risk of developing depression and anxiety. These results align with the theoretical framework of the Self-Regulation Model (SRM), which emphasizes the key role of cognitive and emotional representations in the self-regulation of health problems. This model posits that these representations influence both mental health and overall quality of life.

The negative correlation between treatment control and depression has been supported by several studies (28–31). One study on the general population’s perception of COVID-19, which utilized the same assessment tool as in this study, further reinforced this relationship (29). The study found that greater confidence in the efficacy of current treatments served as a protective factor against depression, as indicated by the negative correlation between treatment control and depressive symptoms. Enhancing public awareness of the effectiveness of available treatments during the pandemic may strengthen the confidence of individuals and improve mental health outcomes based on these findings.

Although few studies have examined the relationship between the timeline cyclical dimension of illness perception and depression or anxiety, the available evidence supports the results of this study. For instance, a validation study of the IPQ-R among Chinese patients with nasopharyngeal cancer demonstrated a significant positive correlation between the perception of cyclical symptoms and anxiety (31). Similarly, research on the relationship between illness perception, depression, and physical activity levels found that timeline cyclical was a predictive risk factor of depression (21). Another study involving patients with chronic rheumatic diseases, though not focused on the direct relationship between illness perception and depression or anxiety, revealed that timeline cyclical significantly contributed to negative emotional states (22). Furthermore, this study highlighted a statistically significant mediating effect of rumination on the relationship between timeline cyclical and negative emotions.

Given that rumination can be seen in some patients with schizophrenia, it is hypothesized that the schizophrenia patients who believe COVID-19 is likely to recur may engage in repeated, passive contemplation of the pain associated with the disease. This may increase their susceptibility to depression and anxiety.

Patients with schizophrenia are a mentally vulnerable population during the COVID-19 pandemic. They showed higher levels of loneliness and depression compared with the general population (32), and depression is in turn a strong predictor of poorer outcomes in schizophrenia (33). According to this study, illness perception concerning COVID-19 significantly influences the severity of depression and anxiety in individuals with schizophrenia. Additionally, illness perception is largely a subjective experience and is amenable to modification (34). So our study suggest that clinicians could contribute to alleviating depression and thereby improve their outcomes during a pandemic by routinely assessing and challenging nonadaptive illness perceptions.

This study has several limitations. First, as a preliminary exploratory survey, it did not establish causal relationships between variables. It is possible that depression and anxiety may influence illness perception rather than the other way around. Second, although the included participants were in a stable condition, their cognitive functioning and perceptions may still have been affected by their illness. Additionally, the limited sample size of the study and the constraints of the study site and time frame may have introduced biases into the findings.

To improve the understanding of this topic, future research should adopt a longitudinal design involving larger, multi-center sample groups. Including a control group without schizophrenia would also provide for more comprehensive insights into the relationship between illness perception and depression/anxiety.

## 5 Conclusion

In conclusion, the perception of illness related to COVID-19 serves as a significant predictor of the emotional state of individuals with schizophrenia. Integrating interventions aimed at addressing and correcting negative illness perceptions into routine health education for this population may help modify maladaptive beliefs, thereby alleviating symptoms of depression and anxiety. This approach enhances adherence to recommended preventive measures during the pandemic, ultimately supporting better mental and physical health outcomes.

## Data Availability

The original contributions presented in the study are included in the article/supplementary material, further inquiries can be directed to the corresponding author.

## References

[1] Mansueto G Palmieri S Marino C Caselli G Sassaroli S Ruggiero GM. The Italian COVID-19 anxiety syndrome scale: investigation of the COVID-19 anxiety syndrome and its association with psychological symptoms in an Italian population. Clin Psychol Psychother. (2022) 29:1972–90. doi: 10.1002/cpp.2767, PMID: 35771682 PMC9350361

[2] Alhakami A Salem V Alateeq D Nikčević AV Marci T Palmieri S. The Arab COVID-19 anxiety syndrome scale (C-19ASS): COVID-19 anxiety syndrome and psychological symptoms in the Saudi Arabian population. Clin Psychol Psychother. (2023) 30:1083–94. doi: 10.1002/cpp.2860, PMID: 37183315

[3] Mansueto G Lopes FL Grassi L Cosci F. Impact of COVID-19 outbreak on Italian healthcare workers versus general population: results from an online survey. Clin Psychol Psychother. (2021) 28:1334–45. doi: 10.1002/cpp.2644, PMID: 34255890 PMC8426916

[4] Brailovskaia J Cosci F Mansueto G Miragall M Herrero R Baños RM. The association between depression symptoms, psychological burden caused by Covid-19 and physical activity: an investigation in Germany, Italy, Russia, and Spain. Psychiatry Res. (2021) 295:113596. doi: 10.1016/j.psychres.2020.113596, PMID: 33261924 PMC7688416

[5] Fisicaro F Lanza G Concerto C Rodolico A di Napoli M Mansueto G. COVID-19 and mental health: a "pandemic within a pandemic". Adv Exp Med Biol. (2024) 1458:1–18. doi: 10.1007/978-3-031-61943-4_1, PMID: 39102186

[6] Chen S Sun X Zhu Q Zhao Y Tang J Song H. Factors influencing the level of depression and anxiety of community-dwelling patients with schizophrenia in China during the COVID-19 pandemic. Int J Environ Res Public Health. (2023) 20:4376. doi: 10.3390/ijerph20054376, PMID: 36901386 PMC10001627

[7] Cheng X Huang X Wu X Lin S. Impact of the COVID-19 pandemic on lifestyle and mental state in patients with schizophrenia: a retrospective study. Medicine. (2023) 102:e32830. doi: 10.1097/MD.0000000000032830 36749244 PMC9901953

[8] Weinman J Petrie KJ. Illness perceptions: a new paradigm for psychosomatics? J Psychosom Res. (1997) 42:113–6. doi: 10.1016/S0022-3999(96)00294-2, PMID: 9076639

[9] Leventhal H Phillips LA Burns E. The common-sense model of self-regulation (CSM): a dynamic framework for understanding illness self-management. J Behav Med. (2016) 39:935–46. doi: 10.1007/s10865-016-9782-2 27515801

[10] Leventhal H. Nerenz D.R. Purse J., Illness Representations and Coping With Health Threats. (1984):219–252.

[11] Weinman J Petrie KJ Moss-morris R Horne R. The illness perception questionnaire: a new method for assessing the cognitive representation of illness. Psychol Health. (1996) 11:431–45.

[12] Brzoska P Yilmaz-Aslan Y Sultanoglu E Sultanoglu B Razum O. The factor structure of the Turkish version of the revised illness perception questionnaire (IPQ-R) in patients with diabetes and cardiovascular disease. BMC Public Health. (2012) 12:852. doi: 10.1186/1471-2458-12-852, PMID: 23043425 PMC3491031

[13] Ashley L Smith AB Keding A Jones H Velikova G Wright P. Psychometric evaluation of the revised illness perception questionnaire (IPQ-R) in cancer patients: confirmatory factor analysis and Rasch analysis. J Psychosom Res. (2013) 75:556–62. doi: 10.1016/j.jpsychores.2013.08.005 24290046

[14] Kim Y Evangelista LS Phillips LR Pavlish C Kopple JD. Racial/ethnic differences in illness, perceptions in minority patients undergoing maintenance hemodialysis. Nephrol Nurs J. (2012) 39:39–48. doi: 10.1016/j.colegn.2011.12.003 PMID: 22480051 PMC3390251

[15] Moss-Morris R Weinman J Petrie K Horne R Cameron L Buick D. The revised illness perception questionnaire (IPQ-R). Psychol Health. (2002) 17:1–16. doi: 10.1080/08870440290001494

[16] Huang W Zhang L Yan J. Psychometric evaluation of the Chinese version of the revised illness perception questionnaire for breast cancer-related lymphedema. Eur J Cancer Care (Engl). (2019) 28:e12900. doi: 10.1111/ecc.12900, PMID: 30144206

[17] Liu Y Wei M Guo L Guo Y Zhu Y He Y. Association between illness perception and health behaviour among stroke patients: the mediation effect of coping style. J Adv Nurs. (2021) 77:2307–18. doi: 10.1111/jan.14761, PMID: 33481272

[18] Yu Y Wu AMS Wing YK Chan JWY Lau MMC Lau JTF. Validation of the revised illness perception questionnaire of obstructive sleep apnea among elderly Chinese in the general population. Sleep Breath. (2023) 27:337–44. doi: 10.1007/s11325-022-02598-y, PMID: 35377089

[19] Yuan J Cao B Zhang C Chan PSF Xin M Fang Y. Changes in compliance with personal preventive measures and mental health status among Chinese factory workers during the COVID-19 pandemic: an observational prospective cohort study. Front Public Health. (2022) 10:10. doi: 10.3389/fpubh.2022.831456 PMC8960195 35359764

[20] Marcil MJ Houchi C Nadarajah K Khairy P Mageau GA Marin MF. The influence of illness perception and coping on anxiety in adults with congenital heart disease. JACC Adv. (2023) 2:100425. doi: 10.1016/j.jacadv.2023.100425, PMID: 38939437 PMC11198082

[21] Nah R Robertson N Niyi-Odumosu FA Clarke AL Bishop NC Smith AC. Relationships between illness representations, physical activity and depression in chronic kidney disease. J Ren Care. (2019) 45:74–82. doi: 10.1111/jorc.12274, PMID: 30938078

[22] Lu Y Jin X Feng LW Tang CSK Neo M Ho RC. Effects of illness perception on negative emotions and fatigue in chronic rheumatic diseases: rumination as a possible mediator. World J Clin Cases. (2022) 10:12515–31. doi: 10.12998/wjcc.v10.i34.12515, PMID: 36579115 PMC9791537

[23] Hagger MS Orbell S. A meta-analytic review of the common-sense model of illness representations. Psychol Health. (2003) 18:141–84. doi: 10.1080/088704403100081321

[24] Hagger MS Koch S Chatzisarantis NLD Orbell S. The common sense model of self-regulation: Meta-analysis and test of a process model. Psychol Bull. (2017) 143:1117–54. doi: 10.1037/bul0000118, PMID: 28805401

[25] Xong NN. Cross-culture adaptation,validity and reliability of the Chinese version of the illness perception questionnaire-revised(IPQ-R) among outpatients. Chin Ment Health J. (2018) 32:713–9. doi: 10.3969/j.issn.1000-6729.2018.09.001

[26] Maguire PA Reay RE Looi JCL. Nothing to sneeze at - uptake of protective measures against an influenza pandemic by people with schizophrenia: willingness and perceived barriers. Australas Psychiatry. (2019) 27:171–8. doi: 10.1177/1039856218815748, PMID: 30501496 PMC7227129

[27] van den Broek-Altenburg E Atherly A. Adherence to COVID-19 policy measures: behavioral insights from the Netherlands and Belgium. PLoS One. (2021) 16:e0250302. doi: 10.1371/journal.pone.0250302, PMID: 34048441 PMC8162620

[28] Feng C Yu B Fu Y Reinhardt JD Yang S. Relationships of illness perceptions with depression and anxiety in people who live with HIV/AIDS in a high-prevalence ethnic autonomous region of Sichuan. China Aids Behav. (2022) 26:3783–93. doi: 10.1007/s10461-022-03707-4, PMID: 35672554

[29] Skapinakis P Bellos S Oikonomou A Dimitriadis G Gkikas P Perdikari E. Depression and its relationship with coping strategies and illness perceptions during the COVID-19 lockdown in Greece: a cross-sectional survey of the population. Depress Res Treat. (2020) 2020:1–11. doi: 10.1155/2020/3158954, PMID: 32908697 PMC7450302

[30] Maltinsky W Henton S Spaltro G Fowler S Chaudhuri R Higgs C. Illness perceptions, symptom severity and psychosocial outcomes in adults with dysfunctional breathing. J Asthma. (2024) 62:226–35. doi: 10.1080/02770903.2024.2397656, PMID: 39212316

[31] Cai Y Zhang Y Cao W Guo VY Deng Y Luo L. Preliminary validation of the revised illness perception questionnaire for patients with nasopharyngeal carcinoma in China. Health. (2023) 11:2469. doi: 10.3390/healthcare11182469, PMID: 37761666 PMC10530590

[32] Lee YR Chung YC Kim JJ Kang SH Lee BJ Lee SH. Effects of COVID-19-related stress and fear on depression in schizophrenia patients and the general population. Schizophrenia (Heidelb). (2022) 8:15. doi: 10.1038/s41537-022-00213-3, PMID: 35249110 PMC8897617

[33] Upthegrove R Marwaha S Birchwood M. Depression and schizophrenia: cause, consequence, or trans-diagnostic issue? Schizophr Bull. (2017) 43:240–4. doi: 10.1093/schbul/sbw097, PMID: 27421793 PMC5605248

[34] Lowe R Norman P. Information processing in illness representation: implications from an associative-learning framework. Health Psychol. (2017) 36:280–90. doi: 10.1037/hea0000457, PMID: 27929331

